# MAD1: Kinetochore Receptors and Catalytic Mechanisms

**DOI:** 10.3389/fcell.2018.00051

**Published:** 2018-05-07

**Authors:** Yibo Luo, Ejaz Ahmad, Song-Tao Liu

**Affiliations:** Department of Biological Sciences, University of Toledo, Toledo, OH, United States

**Keywords:** mitosis, mitotic checkpoint, kinetochore, MAD1, MAD2, protein conformation

## Abstract

The mitotic checkpoint monitors kinetochore-microtubule attachment, delays anaphase onset and prevents aneuploidy when unattached or tensionless kinetochores are present in cells. Mitotic arrest deficiency 1 (MAD1) is one of the evolutionarily conserved core mitotic checkpoint proteins. MAD1 forms a cell cycle independent complex with MAD2 through its MAD2 interaction motif (MIM) in the middle region. Such a complex is enriched at unattached kinetochores and functions as an unusual catalyst to promote conformational change of additional MAD2 molecules, constituting a crucial signal amplifying mechanism for the mitotic checkpoint. Only MAD2 in its active conformation can be assembled with BUBR1 and CDC20 to form the Mitotic Checkpoint Complex (MCC), which is a potent inhibitor of anaphase onset. Recent research has shed light on how MAD1 is recruited to unattached kinetochores, and how it carries out its catalytic activity. Here we review these advances and discuss their implications for future research.

## Introduction

**Figure 1:**
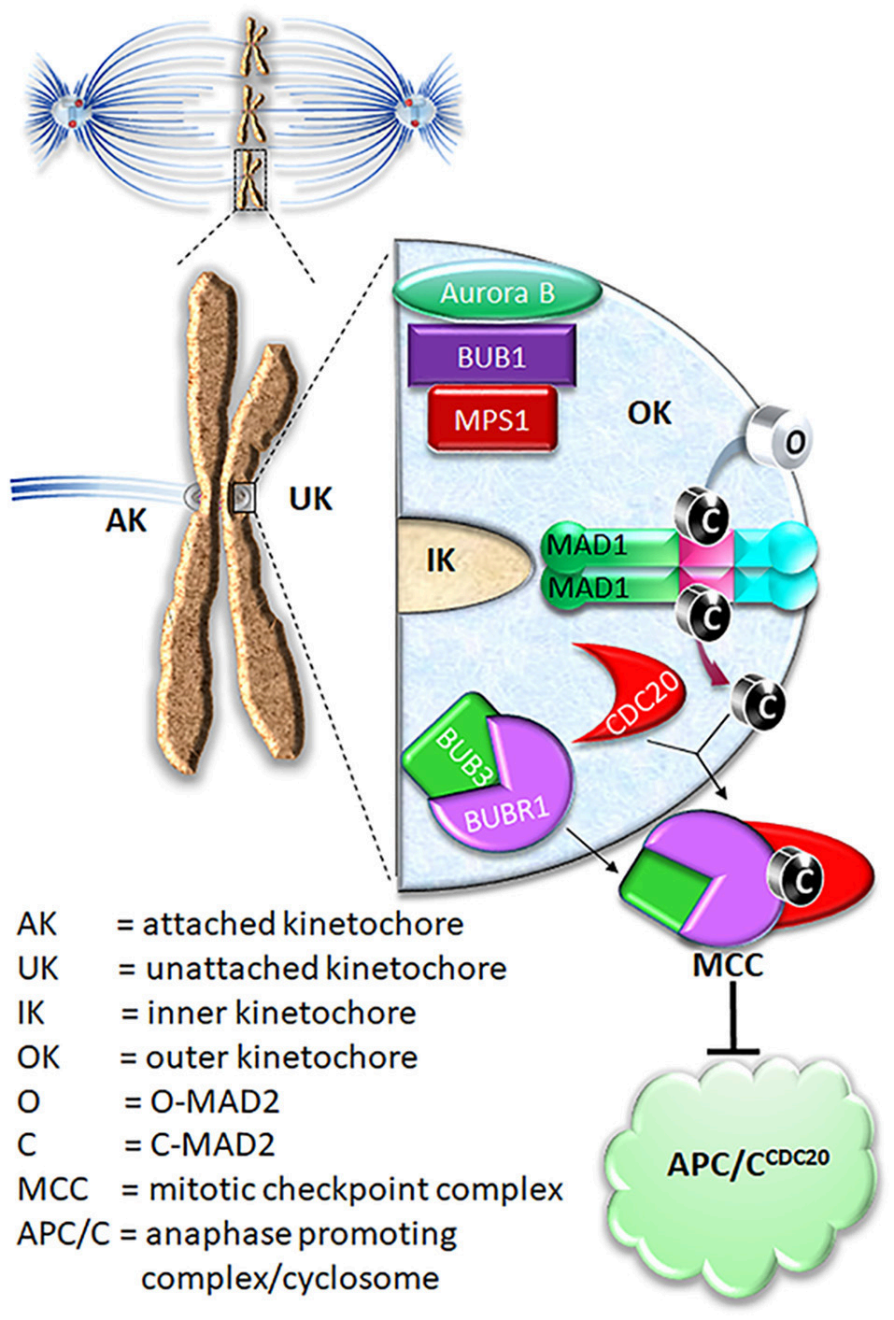
Current model on MAD2 O-C conversion and MCC assembly. In prometaphase, unattached kinetochores initiate mitotic checkpoint signaling, leading to assembly of the mitotic checkpoint complex (MCC) consisting of BUBR1, CDC20, BUB3, and MAD2. The MCC inhibits APC/C ubiquitination activity until all chromosomes are correctly attached to microtubules. For MCC assembly, MAD2 needs to be converted from O conformation into C conformation.

The mitotic checkpoint (or spindle checkpoint or spindle assembly checkpoint) is a crucial mechanism to maintain chromosomal stability. It functions during every prometaphase, detecting the lack of microtubule occupancy or tension at kinetochores and delaying anaphase onset for error correction and faithful chromosome segregation (Lara-Gonzalez et al., 2012; Foley and Kapoor, 2013; Jia et al., 2013; London and Biggins, 2014b; Musacchio, 2015; Liu and Zhang, 2016) (Figure 1). Defects in the mitotic checkpoint may lead to cancer (Weaver and Cleveland, 2007). Sustained mitotic checkpoint triggered by microtubule-targeted cancer drugs or other small molecule inhibitors in clinical trials, on the other hand, causes prolonged mitotic arrest and cancer cell death (Liu and Yen, 2008). Understanding how the mitotic checkpoint works is of significance not only for the fundamental biological question of cell division, but also for improving cancer therapy.

*Mitotic arrest deficiency 1 (MAD1)* is one of the core mitotic checkpoint genes first identified in *S. cerevisiae* and is evolutionarily conserved among most eukaryotic cells (Li and Murray, 1991; Vleugel et al., 2012). Insight into the mechanistic underpinnings of MAD1 function in the mitotic checkpoint has accumulated steadily, with several exciting results obtained in the past few years, yet there are still significant gaps on its working mechanisms. In this review we discuss recent results and potential future directions on MAD1 research. We mostly focus on research in human cells and on two questions: what is the kinetochore receptor for MAD1 and how MAD1 helps MAD2 conformational change.

## Current model on MAD1 functioning mechanism

Shortly after identification of the core mitotic checkpoint genes, their protein products were found to be concentrated at the unattached or tensionless kinetochores, connecting the genetic and cellular aspects of the mitotic checkpoint control mechanisms (Chen et al., 1996; Waters et al., 1998). MAD1, MAD2, and MPS1 levels drop below detection at attached kinetochores, indicating their sensitivity to the kinetochore-microtubule attachment status. It was later established that the major function of MAD1 is to recruit MAD2 to unattached kinetochores, where MAD2 molecules are converted from open (O-MAD2) to closed (C-MAD2) conformation. The conformational change of MAD2 is a critical signal amplification mechanism for the mitotic checkpoint (Mapelli and Musacchio, 2007; Luo and Yu, 2008).

O-MAD2 is the predominant conformer in interphase cells (Luo et al., 2004; Fava et al., 2011). In the current model, a 2:2 MAD1:C-MAD2 tetramer localized at unattached kinetochores functions as the catalyst for MAD2 O-C conversion, initiating the mitotic checkpoint response when cells enter mitosis (De Antoni et al., 2005). It was suggested that O-MAD2 hetero-dimerizes with the C-MAD2 subunit in the MAD1:C-MAD2 tetramer and converts into C-MAD2 (Mapelli and Musacchio, 2007; Luo and Yu, 2008). The mechanism of conversion is unknown but possibly involves one or more intermediate MAD2 conformations (I-MAD2) (Mapelli and Musacchio, 2007; Luo and Yu, 2008; Hara et al., 2015). C-MAD2 conformation is required to interact with BUBR1 and CDC20 to form the Mitotic Checkpoint Complex (MCC), which binds and inhibits the anaphase promoting complex/cyclosome (APC/C^CDC20^) (Lara-Gonzalez et al., 2012; London and Biggins, 2014b; Musacchio, 2015; Liu and Zhang, 2016) (Figure 1). As the E3 ubiquitin ligase activity of the APC/C^CDC20^ is inhibited by the MCC, cells are arrested at the metaphase. It is important to note that the CDC20 molecule associated with the APC/C is a substrate-binding and activator subunit for the APC/C, and is separate from the second CDC20 molecule as a subunit of the MCC (Izawa and Pines, 2015; Alfieri et al., 2016; Yamaguchi et al., 2016). C-MAD2 in cells may also directly bind to free CDC20, blocking its association with the APC/C core thus keeping APC/C activity low (Fang et al., 1998; Izawa and Pines, 2012).

## Structure of MAD1

**Figure 2:**
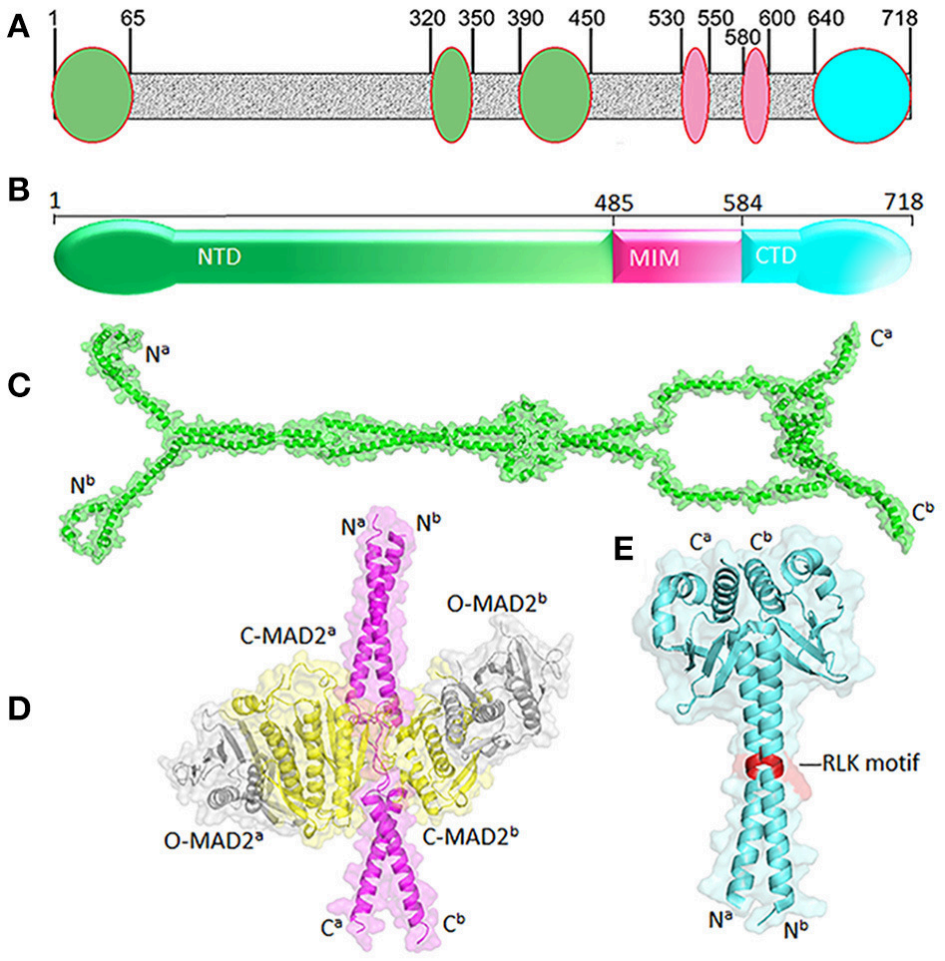
Structural features of MAD1. **(A)** Non-coiled coil segments (ovals) are scatted along likely coiled-coil regions of MAD1 (in gray) as predicted by COILS program (Lupas et al., 1991). **(B)** Diagram of MAD1 showing its N-terminal domain (NTD), the domain containing the MAD2 interaction motif (MIM) and C-terminal domain (CTD). **(C)** Predicted model of MAD1^NTD^ dimer by Galaxy Homomer (Baek et al., 2017). **(D)** Crystal structure of MAD1^MIM^ (pink) dimer bound with two molecules of C-MAD2 (yellow) as in PDB 1GO4 (Sironi et al., 2002). Two O-MAD2 molecules (gray) from 2V64 (Mapelli et al., 2007) are also fitted. **(E)** Crystal structure of MAD1^CTD^ dimer from 4DZO (Kim et al., 2012). The RLK motifs are shown in red. Superscripts a&b denote two different chains of the same molecule. PyMol was used for structure visualization and model generation.

Human MAD1 is a protein of 718 amino acids (Figure 2). The structure of full length MAD1 has not been solved yet, mainly due to low solubility of recombinant MAD1, although Faesen et al. recently reported purification of full-length MAD1 (co-expressed with MAD2) from *Tnao*38 insect cells (Faesen et al., 2017). MAD1 is often simplified as a coiled coil molecule along its entire length, but structural analyses and predictions indicated several non-coiled coil segments interrupting the coiled coil regions (Lupas et al., 1991) (Figure 2A). The N-terminal domain of MAD1 (1–485 residues, MAD1^NTD^) is thought to target the protein to nuclear envelope or kinetochores, is required for MAD1 dimerization and interacts with many other proteins (Chen et al., 1998; Martin-Lluesma et al., 2002; Rodriguez-Bravo et al., 2014; Akera et al., 2015; Ji et al., 2018). A predicted structure of MAD1^NTD^ is presented in Figure 2C. The best characterized MAD1 domain contains its MAD2 interaction motif (MIM, 485–584 residues). The solved crystal structure of MAD1^MIM^ in complex with C-MAD2 is the cornerstone for the now classical model of the MAD1:C-MAD2 catalyst (Sironi et al., 2002). In the structure, MAD1^MIM^ forms a dimer, with each monomer utilizing the disordered loop spanning 530–550 residues to trap one molecule of C-MAD2 and assemble a 2:2 heterotetramer (Figure 2D) (Sironi et al., 2002). Although the MIM motif was suggested to be absent in MAD1 homologs in *Salpingoeca rosetta* (a choanoflagellate), *Micromonas pusilla* (a green algae), and *Naegleria gruberi* (an amoeboflagellate) (Vleugel et al., 2012), the particular sequence alignments need to be treated more cautiously as the annotated MAD1s might only be partial sequences from genome projects. The C-terminal domain of MAD1 (585–718 residues, MAD1^CTD^) is also evolutionarily conserved and many results have confirmed its functional importance in maintaining the mitotic checkpoint (Chen et al., 1999; De Antoni et al., 2005; Yang et al., 2008; Ballister et al., 2014; Heinrich et al., 2014; Kruse et al., 2014; Kuijt et al., 2014; Ji et al., 2018). The N-terminal portion of MAD1^CTD^ adopts an α-helical structure and forms a coiled coil stem (597–637 residues) while its C-terminal (638–718) residues fold into a globular domain (Figure 2E). The overall MAD1^CTD^ dimer structure resembles the kinetochore-binding domains of Spc25 and Csm1 or the RWD domain (Kim et al., 2012).

## Kinetochore receptors of MAD1

MAD1 forms a cell cycle independent complex with MAD2. The yeast MAD1:MAD2 complex is stable enough to tolerate harsh conditions such as 5M NaCl and 1M Urea (Chen et al., 1999). The observed stable complex most likely reflects C-MAD2 bound to MAD1^MIM^, as point mutations at the MAD1^MIM^ abolished most of MAD2 signals in both imaging and immunoprecipitation experiments (Sironi et al., 2002; Maldonado and Kapoor, 2011; Ji et al., 2018). Such MAD1:C-MAD2 complexes are localized to the nuclear envelope during interphase, coinciding with a fraction of MPS1, and have been shown to generate a basal level of C-MAD2 in cells that contributes to the mitotic checkpoint (Chen et al., 1998; Campbell et al., 2001; Liu et al., 2003a; Lee et al., 2008; Rodriguez-Bravo et al., 2014). However, it is generally accepted that C-MAD2 production peaks during prometaphase, when the MAD1:C-MAD2 complex is localized to the unattached kinetochores (Chen et al., 1998; Tipton et al., 2011a). Therefore, although the interaction between MAD1^MIM^ and C-MAD2 does not change in interphase and prometaphase cells, some mitosis-specific modifications or interactions must have occurred to the MAD1:C-MAD2 tetramer, the catalyst for the MAD2 O to C conformation conversion reaction, to make it more efficient in generating C-MAD2. To better understand the enhanced catalytic activity of the MAD1:C-MAD2 complex in prometaphase cells, identifying its kinetochore receptor(s) is certainly an important question.

**Figure 3:**
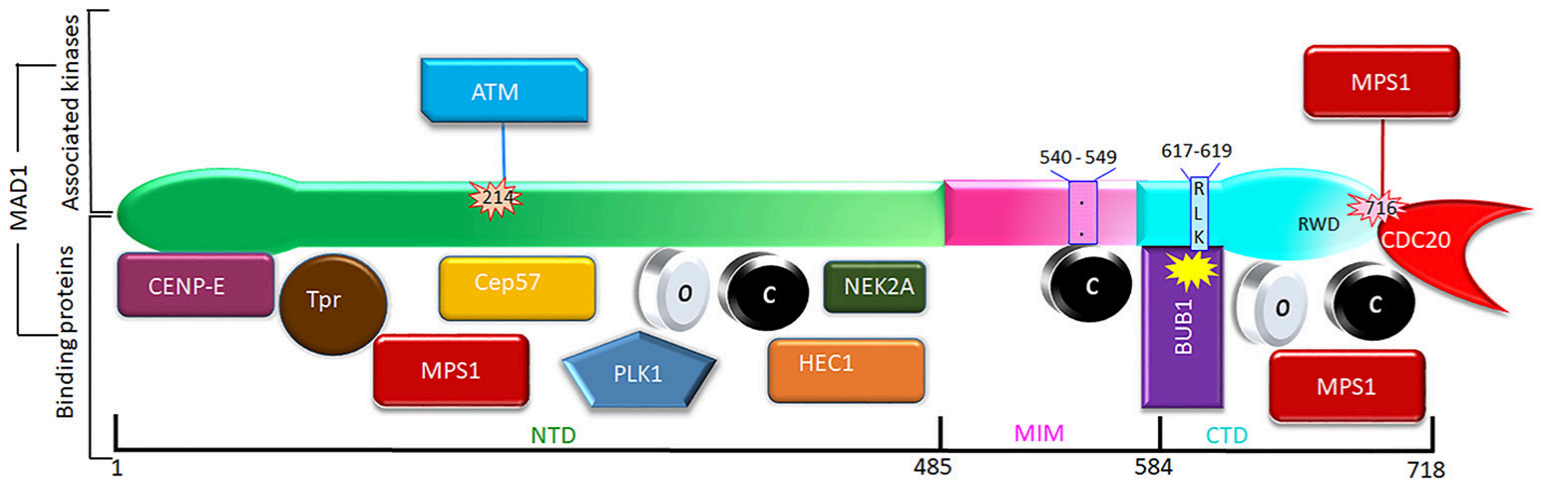
Phosphorylation (above) and interacting partners (below) of MAD1. Only the phosphorylation events and interactions with better-defined functional implications are shown. The interacting proteins are roughly grouped based on their binding to the NTD, MIM, or CTD of MAD1.

In earlier efforts to characterize kinetochore receptor(s) for MAD1, people have tried to refine the region in MAD1 for kinetochore targeting and determine the dependency relationship of MAD1 to various proteins for kinetochore localization. Biochemical assays have also uncovered multiple MAD1 interacting proteins (Figure 3). MAD1^NTD^ was long thought to be responsible for its kinetochore localization (Chung and Chen, 2002). However, MAD1^CTD^ also plays a role in MAD1 kinetochore localization (Kim et al., 2012). Based on epistatic analysis using siRNAs, MAD1 kinetochore localization was mapped into a CENP-I
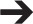
NDC80
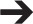
MPS1
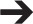
MAD1
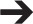
MAD2 dependency hierarchy (Martin-Lluesma et al., 2002; Liu et al., 2003b, 2006; Matson and Stukenberg, 2014). Other results concluded that ROD and ZW10 are also required for MAD1 localization (Kops et al., 2005). Additional knockdown and knockout based analyses, together with genetic mutants in model organisms, have provided clues to the requirement of other proteins (including BUB1) for MAD1 kinetochore localization (Johnson et al., 2004; Meraldi and Sorger, 2005; Liu et al., 2006; Qian et al., 2017). Biochemically, MAD1^NTD^ interacts with NDC80 (Martin-Lluesma et al., 2002), PLK1 (Chi et al., 2008), NEK2 (Lou et al., 2004), TPR (Lince-Faria et al., 2009), CEP57 (Zhou et al., 2016), and CENP-E (Akera et al., 2015), while MAD1^CTD^ binds directly to BUB1 as well as CDC20 (Ji et al., 2017) (Figure 3). Our recent work has shown that both the NTD and CTD of MAD1 interact with MPS1 and that MAD1 has additional binding sites for MAD2 outside the MIM region (Ji et al., 2018). We next discuss MAD1 interacting proteins as potential kinetochore receptors for the MAD1:C-MAD2 complex.

### BUB1 as a kinetochore receptor of MAD1

In budding yeast, an RLK motif in the MAD1^CTD^ was long known to direct interaction with BUB1 when the mitotic checkpoint is activated (Brady and Hardwick, 2000). London et al. demonstrated that BUB1 is phosphorylated in the middle region by MPS1 kinase, and that the phosphorylated region then binds to MAD1 and recruits it to kinetochores (London and Biggins, 2014a). Except in *C. elegans* (Moyle et al., 2014), the RLK motif in MAD1 and the corresponding region in BUB1 (conserved domain 1 or CD1) are evolutionarily conserved, and the RLK motif is essential for MAD1 kinetochore targeting in fission yeast and human cells (Klebig et al., 2009; Kim et al., 2012; Heinrich et al., 2014; Mora-Santos et al., 2016). Puzzlingly, direct interaction between BUB1 and MAD1 was not observed in human cell lysates (Kim et al., 2012). However, recently several labs have confirmed that human BUB1 is phosphorylated at S459 by CDK1, and then further phosphorylated by MPS1 at T461. The doubly phosphorylated BUB1 directly binds to the RLK motif in MAD1 and recruits MAD1 to kinetochores (Faesen et al., 2017; Ji et al., 2017; Zhang et al., 2017). It should be noted that targeting of BUB1 to the kinetochore also requires MPS1 activity. Specifically, MPS1 phosphorylates the outer kinetochore protein KNL1 at threonine (T) residues of its “MELT” repeat motifs. The phosphorylated MELT motifs are recognized by BUB3, which in turn recruits its binding partner BUB1 to kinetochores (London et al., 2012; Shepperd et al., 2012; Yamagishi et al., 2012; Primorac et al., 2013; Vleugel et al., 2013; Krenn et al., 2014; Zhang et al., 2014).

Intriguingly, Qian et al recently reported that the BUB1:MAD1 interaction only occurs transiently in early prometaphase in unperturbed mitosis (Qian et al., 2017). During prolonged prometaphase after cells were challenged by microtubule drugs nocodazole and taxol or the Eg5 kinesin inhibitor STLC, MAD1 levels at unattached kinetochores remained high, even in the absence of BUB1 (Qian et al., 2017). These results agreed with earlier observations implying BUB1-independent mechanisms for MAD1 kinetochore localization. For example, in both fission yeast and *Drosophila*, MELT-phosphomimetic KNL1 mutants are sufficient to retain BUB1 but not MAD1 at kinetochores (Shepperd et al., 2012; Vleugel et al., 2012).

### The ROD/ZW10/ZWILCH complex as a kinetochore receptor for MAD1

The ROD/ZW10/ZWILCH or RZZ complex are kinetochore proteins found only in metazoans (Karess, 2005). Multiple studies showed that the RZZ complex is required for MAD1 and MAD2 localization at kinetochores and mitotic checkpoint signaling under all treatment conditions (Basto et al., 2000; Chan et al., 2000; Buffin et al., 2005; Kops et al., 2005; Silió et al., 2015; Qian et al., 2017). More recent data supported that a pool of RZZ requires the N-terminal region of KNL1 for kinetochore localization in BUB1-dependent manner (Caldas et al., 2015; Zhang et al., 2015). Interestingly, the same CD1 domain of BUB1 is required for this pool of RZZ and MAD1 kinetochore localization (Zhang et al., 2015, 2017). As mentioned above, BUB1 might not be the only kinetochore receptor for MAD1, but MAD1 kinetochore localization always requires RZZ (Qian et al., 2017). There is another pool of RZZ whose localization is dependent neither on KNL1 nor on BUB1, but possibly on NDC80 that is associated with CENP-T (Caldas et al., 2015; Samejima et al., 2015). It awaits to be further studied whether this pool of RZZ retains MAD1 at kinetochores during nocodazole treatment when the BUB1:MAD1 interaction is lost (see BUB1 as a Kinetochore Receptor of MAD1). While MAD1 is found in RZZ immunoprecipitates, direct interaction between MAD1 and the RZZ complex subunits remains to be established (Défachelles et al., 2015).

### NDC80 as a kinetochore receptor for MAD1

NDC80 is a subunit of the NDC80/NUF2/SPC24/SPC25 complex, whose microtubule binding activity is primarily responsible for end-on attachment of microtubules to kinetochores (Santaguida and Musacchio, 2009). Depletion of NDC80 and NUF2 caused mis-localization of MAD1:MAD2 complex and defective checkpoint activation (Meraldi et al., 2004). Depletion of SPC25 also resulted in loss of NDC80 and MAD1 from kinetochores, but had no impact on BUB1 levels at kinetochores (Bharadwaj et al., 2004). These results lent support to the notion that MAD1 has one or more kinetochore receptors that involve the NDC80 complex. Yeast two-hybrid assay showed NDC80 interacts with MAD1^NTD^ (Martin-Lluesma et al., 2002), but the interaction has not been observed by immunoprecipitation in cell lysates. However, immunoprecipitation in cell lysates might not be a gold standard in assessing protein-protein interactions at kinetochores, where proteins are normally enriched to much higher concentrations than in the cytosol, are spatially proximal to multiple proteins, and may undergo particular posttranslational modifications such as phosphorylation. In addition, the concentration of any particular endogenous protein in a cell lysate at 1 mg/ml total protein level can be 10~1,000 times lower than its intracellular level. These factors might cause failure in detecting a physiologically relevant protein-protein interaction such as the MPS1:NDC80 interaction by immunoprecipitation using cell lysates (Aravamudhan et al., 2015; Hiruma et al., 2015; Ji et al., 2015). Testing protein-protein interactions using recombinant proteins *in vitro* and immunofluorescence of protein mutants in cells could complement the results from yeast two-hybrid and immunoprecipitation using cell lysates. It remains to be characterized whether and how direct MAD1 interaction with NDC80 could occur at kinetochores, and whether MAD1-NDC80 interaction could serve as a microtubule attachment sensitive checkpoint activation mechanism.

### CEP57 as a kinetochore receptor for MAD1

Cep57 is a microtubule binding protein that localizes at centrosomes and kinetochores in *Xenopus* and human cells (Emanuele and Stukenberg, 2007; Zhou et al., 2016). Zhou et al. showed that Cep57 kinetochore localization depends on its interaction with Mis12 of the KMN network through its N-terminal region (Zhou et al., 2016). Cep57 also helps recruit MAD1 and MAD2 to kinetochores. The recruitment is mediated by direct interaction between MAD1 and the Cep57 C-terminal region which overlaps with its microtubule binding domain. Interestingly, the microtubule binding of Cep57 at kinetochores competitively displaces MAD1, suggesting a mechanism to couple MAD1 localization with microtubule attachment status at kinetochores (Zhou et al., 2016). However, the mitotic checkpoint defects in Cep57 depleted cells are much weaker compared to MAD1 or MAD2 depletion, again suggesting the presence of other proteins in recruiting MAD1 to unattached kinetochores.

### Consideration of other proteins as potential kinetochore receptor for MAD1

Kinetochores as a platform may provide a MAD1 recruitment interface that is contributed by multiple distinct proteins, and the interface may dynamically change at different cell cycle stages or when cells are exposed to different drugs. Individual interactions might be weak, but the collective force can be both strong and highly specific (Kim et al., 2012). In the literature, there are many other proteins shown to affect MAD1 kinetochore localization. Most of them may work indirectly through affecting the MAD1 receptors discussed above, but it is also possible some might directly contribute to kinetochore localization of MAD1. We briefly comment on some of these proteins (Figure 3).

Nek2 kinase interacts with MAD1, but it is degraded during prometaphase, so the significance of its interaction with MAD1 in terms of mitotic checkpoint signaling is unclear (Lou et al., 2004; Hayes et al., 2006; Sedgwick et al., 2013). Similarly, inhibition or knockdown of Plk1 kinase causes strong prometaphase arrest, arguing against the possibility Plk1 is a major kinetochore receptor for the MAD1:C-MAD2 complex, although recent reports suggested that Plk1 might contribute to the mitotic checkpoint by potentiating MPS1 or phosphorylating CDC20 (Sumara et al., 2004; Lénárt et al., 2007; Chi et al., 2008; Liu et al., 2012; Espeut et al., 2015; O'Connor et al., 2015; von Schubert et al., 2015; Jia et al., 2016; Ikeda and Tanaka, 2017). Plk1 and MPS1 share many substrates, and Plk1 even phosphorylates many MPS1 autophosphorylation sites, so Plk1 functions in the mitotic checkpoint in *C. elegans*, which naturally lack a MPS1 homolog (Dou et al., 2011; Espeut et al., 2015; von Schubert et al., 2015). As mentioned, MPS1 was long known to be essential for MAD1 kinetochore localization (Martin-Lluesma et al., 2002; Liu et al., 2003a). In light of recent results, MPS1 may phosphorylate BUB1 to create MAD1 binding sites at kinetochores (Ji et al., 2017; Zhang et al., 2017). We showed that MPS1 binds and phosphorylates both MAD1^NTD^ and MAD1^CTD^ at least *in vitro* (Ji et al., 2018). MAD1 phosphorylation by MPS1 has also been shown by other groups (Faesen et al., 2017; Ji et al., 2017). However the MAD1^CTD^:MPS1 interaction was weakened when MAD1^CTD^ was phosphorylated by MPS1, arguing against the role of MPS1 as the kinetochore receptor for MAD1 (Ji et al., 2018). TPR interacts with MAD1 and MAD2 in both interphase and mitosis, but it is largely dispensable for MAD1 kinetochore localization (Lee et al., 2008; Rodriguez-Bravo et al., 2014). CENP-E interaction with MAD1 was suggested to be involved in chromosome alignment. Whether it helps initiate MAD1 activity for the mitotic checkpoint is unclear (Akera et al., 2015).

## MAD1 in the catalysis of MAD2 O-C conversion and MCC assembly

Although kinetochore localization of MAD1 is important for its regulation and activity, the localization itself is not sufficient for MAD2 O-C conversion and the mitotic checkpoint response (i.e., maintaining metaphase arrest). This principle was first demonstrated in experiments by Maldonado and Kapoor (2011). When MAD1 was fused with centromere protein Mis12 to be constitutively targeted to kinetochores, cells were arrested at metaphase even after all kinetochores were attached by microtubules. However, the arrest was abrogated by inhibitors of MPS1 or Aurora B kinases, suggesting that these conserved kinases affect catalytic functions of MAD1 or other aspects of the mitotic checkpoint signaling (Maldonado and Kapoor, 2011). Using a similar strategy, we and others have shown that removing either the NTD or CTD regions from MAD1 negatively impacted the mitotic checkpoint, most likely due to reduced catalysis of MAD2 O-C conversion and/or MCC assembly (Tipton et al., 2013; Ballister et al., 2014; Heinrich et al., 2014; Kruse et al., 2014; Kuijt et al., 2014; Ji et al., 2018). Further understanding the MAD2 O-C conversion mechanisms requires dissection of contribution of MAD1 dimerization, interactions between different MAD1 domains, and the interplay of MAD1 with different kinases (Figure 3).

### MAD1 dimerization

MAD1 forms a dimer mostly through its coiled coil segments (Figure 1), but whether the dimerization is regulated or is functionally important is largely unknown. Kim et al. identified several mutations in the CTD globular domain that disrupted both homodimerization and kinetochore localization of MAD1 (Kim et al., 2012). ATM, a canonical DNA damage checkpoint protein, mediates S214 phosphorylation that regulates MAD1 dimerization as well as heterodimerization with MAD2, and contributes to mitotic checkpoint function and chromosomal stability (Yang et al., 2014). A recent report suggested that PTEN affects MAD1 dimerization in interphase (Liu et al., 2017). When tested as fusions with Mis12, both MAD1^NTD^ and MAD1^CTD^ are required for mitotic checkpoint signaling (Ji et al., 2018). We showed that both MAD1^CTD^ and MAD1^NTD^ are required for MAD1 dimerization, and MPS1 kinase might regulate MAD1 dimerization (Ji et al., 2018). However, as MAD1^NTD^ and MAD1^CTD^ both have important binding partners, we cannot conclusively ascribe the checkpoint defects caused by MAD1 truncations to defective MAD1 dimerization.

### MAD1 inter-domain interactions

Previously it was reported that MAD1^CTD^ can possibly fold back to be close to the MAD1^MIM^:C-MAD2 catalytic center and the recruited MAD2 molecules undergoing conformational conversion (Sironi et al., 2002). We did not detect direct interaction of recombinant MAD1^MIM^ with either MAD1^NTD^ or MAD1^CTD^ (Ji et al., 2018), arguing against the possibility that the α-helical stem in the CTD forms an anti-parallel coiled coil with a helical portion of MIM (Sironi et al., 2002; Heinrich et al., 2014). This agrees with the current MAD1^CTD^ dimer structure, in which the α-helical portions in two CTD monomers form a coiled coil (Kim et al., 2012). However, studies on the phosphorylation of Thr716 which is two residues away from the C-terminal end, on mutations of several charged residues at the MAD1^CTD^ globular domain, and on mutations along the MAD1^CTD^ α-helical portion (Ser610, or RLK^617−619^, or Tyr634), revealed that MAD1^CTD^ positively contributes to mitotic checkpoint signaling, most likely by promoting MAD2 O-C conversion and/or MCC assembly (see below) (Heinrich et al., 2014; Kruse et al., 2014; Faesen et al., 2017; Ji et al., 2017, 2018). How this regulation occurs is still unclear, but a posttranslational modification-driven CTD fold-back is a possible mechanism.

Interestingly we found that MAD1^NTD^ and MAD1^CTD^ directly interact with each other, and that the interaction is reduced after phosphorylation by MPS1 (Ji et al., 2018). We speculate that relaxing the interaction between MAD1^NTD^ and MAD1^CTD^ elevates the efficiency of the MAD1:C-MAD2 catalyst, and that interphase MAD1 might be in a ground-state conformation. We are still uncertain whether the interaction between MAD1^NTD^ and MAD1^CTD^ occurs within a MAD1 dimer or between MAD1 dimers. Nevertheless, cellular experiments suggested that both NTD and CTD contribute positively to the mitotic checkpoint (Ji et al., 2018).

### MAD1 in MAD2 O-C conversion

As mentioned, the major activity of MAD1 is to catalyze MAD2 O-C conversion during mitotic checkpoint signaling. However, how the MAD1:C-MAD2 heterotetramer catalyzes the O-MAD2 converting into C-MAD2 is largely unknown. In the past two years, several publications have advanced our understanding of the underlying mechanisms. Ji et al. in the Yu lab reported that MAD1 Thr716 phosphorylation by MPS1 helps generate the APC/C inhibitory activity in a series of *in vitro* reconstitution experiments (Ji et al., 2017). Faesen et al. established a FRET assay to detect MAD2 conformational conversion by measuring its interaction with BUBR1 and formation of the MCC, both of which require C-MAD2 (Faesen et al., 2017). They found that MPS1 phosphorylates MAD1 directly, after which MAD1:MAD2 complexes accelerate MAD2 O-C conversion. Moreover, phosphorylation of the MAD1^CTD^ at S699, S713, and T716 by MPS1 is essential for this acceleration. How MAD1^CTD^ and its phosphorylation facilitate the MAD2 O-C conversion was unclear from these studies. Our recently published results suggested at least one mechanism how MAD1^CTD^ and MAD1^NTD^ can assist MAD2 O-C conversion. We found that both MAD1^NTD^ and MAD1^CTD^ directly bind to both O and C conformers of MAD2. We also showed that mutating T716 into alanine significantly reduced the MAD1^CTD^ binding to both C- and O-MAD2 (Ji et al., 2018).

**Figure 4:**
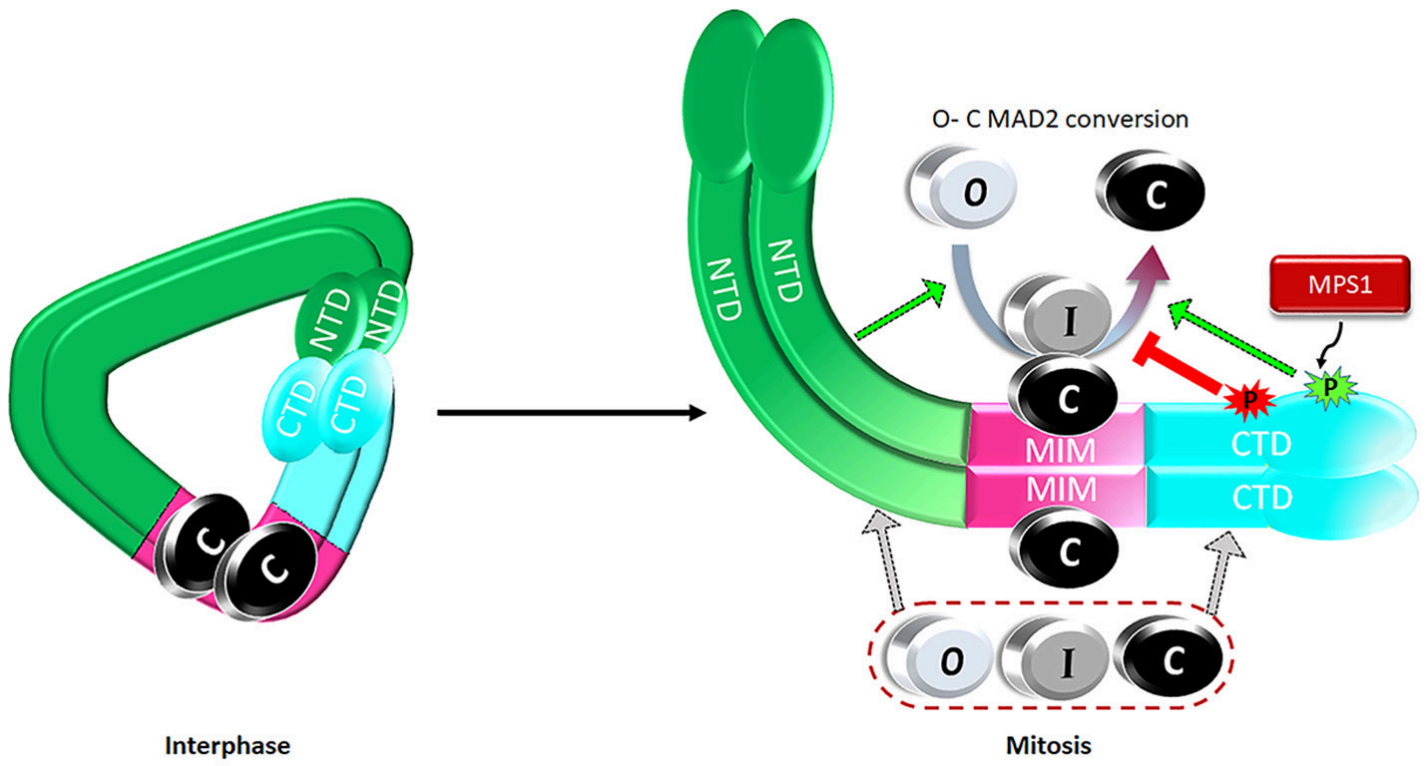
A proposed model on regulation of the catalytic activity of the MAD1:C-MAD2 heterotetramer to drive MAD2 O-C conversion. See text for details.

Expanding the MAD1 functional domains beyond its classical MIM region to NTD and CTD helps address one major puzzle in mitotic checkpoint studies. It is well known that more C-MAD2 is produced when cells enter mitosis (Luo et al., 2004; Fava et al., 2011), but how could this happen if the levels of MAD2 and the assumed catalyst for MAD2 O-C conversion, the stable MAD1^MIM^:C-MAD2 heterotetramer, do not change throughout the cell cycle? We proposed a model that during interphase MAD1^NTD^ and MAD1^CTD^ interact with each other, limiting their binding to O- or C-MAD2 (Figure 4). Despite MAD1^MIM^ binding to the C-MAD2, the MAD1 configuration during interphase is at a ground state that shows only low level catalytic activity for MAD2 O-C conversion. When cells enter mitosis, MPS1 activity relaxes the MAD1^NTD^ and MAD1^CTD^ interaction, and more O-MAD2 is recruited to MAD1, through either the MAD1^NTD^ or MAD1^CTD^. The law of mass action, together with modification at the MAD1^MIM^:C-MAD2 catalytic center and probably also in the MAD1^CTD^, promote more production of C-MAD2 (Figure 4) (Ji et al., 2018). I-MAD2 has been surmised to exist during MAD2 O-C conversion and was shown to share structure similarity to C-MAD2 (Mapelli et al., 2007; Hara et al., 2015). We showed that both MAD1^NTD^ or MAD1^CTD^ bind to at least one form of I-MAD2 (MAD2^Δ*N*10^) (Ji et al., 2018). Such binding may also facilitate MAD2 O-C conversion.

### MAD1 in MCC assembly

Studies on MAD1^NTD^ and MAD1^CTD^ also indicated direct involvement of MAD1 in facilitating MCC assembly, an activity intricately linked with but possibly separated from catalyzing MAD2 O-C conversion. The MCC is assembled from CDC20, C-MAD2 and the cell cycle independent BUBR1:BUB3 subcomplex (Sudakin et al., 2001; Liu and Zhang, 2016). MAD1 at kinetochores may help bring the MCC subunits in spatial proximity to promote MCC assembly. MAD1 phosphorylation at Thr716 by MPS1 kinase was shown to increase its interaction with CDC20 through a basic motif ^27^RWQRK^31^ in CDC20 (Ji et al., 2017). MAD1 at least in prometaphase is recruited to kinetochores by BUB1, which in turn is localized through KNL1 (Caldas et al., 2015; Zhang et al., 2015, 2017; Faesen et al., 2017; Ji et al., 2017). The BUBR1:BUB3 subcomplex can be recruited to KNL1 directly or through interaction with BUB1 (Overlack et al., 2015; Zhang et al., 2016). In addition, both BUB1 and BUBR1 directly interact with CDC20 at kinetochores (Lischetti et al., 2014; Diaz-Martinez et al., 2015; Di Fiore et al., 2015). Together with C-MAD2 produced by the MAD1:C-MAD2 heterotetramer, the spatial proximity of the MCC subunits, guided by protein-protein interactions, presumably increases the efficiency of MCC assembly. Such explanation links MCC production to the unattached kinetochores as the assembly platform. The energy favorable MCC assembly may also be coupled with MAD2 O-C conversion to help overcome the activation energy barrier for MAD2 conformational change (Faesen et al., 2017; Ji et al., 2017, 2018). During MCC assembly, MAD2 uses its “safety belt” structural motif to interact with CDC20, and its “dimerization domain” to interact with BUBR1 (Tipton et al., 2011b; Chao et al., 2012; Alfieri et al., 2016; Yamaguchi et al., 2016). We found that the MAD1^NTD^ or MAD1^CTD^ association with C-MAD2 or I-MAD2 does not require these two known MAD2 motifs (Ji et al., 2018). This may create some advantage to relay newly converted C-MAD2 from MAD1 to the MCC, as those C-MAD2 molecules still have the “safety belt” and the “dimerization domain” readily accessible for MCC assembly.

## Conclusions

As one of the most important checkpoint proteins, MAD1 forms a cell cycle independent MAD1:C-MAD2 complex to catalyze the MAD2 O-C conversion at unattached kinetochores, thus amplifying the signals for the mitotic checkpoint and promoting the formation of the MCC, the potent inhibitor of anaphase onset. Previous research on MAD1 has focused on its MIM domain. The line of work provided tremendous insights into the MAD1 working mechanism but still left many questions, the predominant one being how to reconcile the cell cycle independent MAD1^MIM^:C-MAD2 catalyst formation with cell cycle dependent C-MAD2 production. Recent results suggested that MAD1^NTD^ and MAD1^CTD^ domains may be an integral part of the regulatory mechanisms to control MAD1 activity in promoting MAD2 O-C conversion. In addition, MAD1 may also be part of the scaffold to streamline MCC assembly at kinetochores. In the near future, it is crucial to clarify the kinetochore receptor(s) for MAD1 and to establish how microtubule attachment disrupts the interaction between MAD1 and its kinetochore receptors. It will also be intriguing to further elucidate how different domains of MAD1 respond to modifications by protein kinases such as MPS1, BUB1 and Aurora B, and their counter-acting phosphatases to control MAD2 O-C conversion and MCC assembly. MAD1 demands and deserves more attention.

## Author contributions

YL and EA contribute equally to the manuscript. Both YL and EA drafted the manuscript and made the figures. S-TL revised the whole manuscript critically for important intellectual content. All authors give final approval of the manuscript.

### Conflict of interest statement

The authors declare that the research was conducted in the absence of any commercial or financial relationships that could be construed as a potential conflict of interest.
